# Delivering a Tai Chi Intervention to Adults Aging with Mobility Disabilities Using Zoom

**DOI:** 10.1093/geroni/igab046.1642

**Published:** 2021-12-17

**Authors:** Tracy Mitzner, Elena Remillard, Kara Cohen, Lelah Cochran

**Affiliations:** 1 Georgia Institute of technology, Atlanta, Georgia, United States; 2 Georgia Institute Of Technology, Atlanta, Georgia, United States; 3 Georgia Institute of Technology, Atlanta, Georgia, United States

## Abstract

Many individuals aging with mobility disabilities experience barriers to participating in physical activity, including transportation challenges and the need for specialized instruction. Since the COVID-19 pandemic began, these participation barriers have been amplified due to lockdowns and restrictions. Tele-technologies, including videoconferencing platforms like Zoom, can facilitate access to exercise classes from one’s home. Virtual group exercise classes that incorporate social interaction have particular potential to support the physical and mental health of this population. This session will highlight lessons learned from launching the ‘Tele Tai Chi’ study, in which we are delivering an evidence-based Tai Chi program (Tai Chi for Arthritis) via Zoom to small group classes of older adults with long-term mobility disabilities. We will describe adaptations made in translating the in-person program to an interactive, online class, and provide an overview of a ‘Telewellness’ Tool that provides guidelines for using Zoom to deliver exercise classes to older adults.

